# Naphtha (Erdöl), mit Wasserstoff behandelte schwere: MAK-Begründung, Nachtrag

**DOI:** 10.34865/mb6474248yold10_2ad

**Published:** 2025-06-30

**Authors:** Andrea Hartwig, MAK Commission

**Affiliations:** 1 Institut für Angewandte Biowissenschaften. Abteilung Lebensmittelchemie und Toxikologie. Karlsruher Institut für Technologie (KIT) Adenauerring 20a, Geb. 50.41 76131 Karlsruhe Deutschland; 2 Ständige Senatskommission zur Prüfung gesundheitsschädlicher Arbeitsstoffe. Deutsche Forschungsgemeinschaft, Kennedyallee 40, 53175 Bonn, Deutschland. Weitere Informationen: Ständige Senatskommission zur Prüfung gesundheitsschädlicher Arbeitsstoffe | DFG

**Keywords:** Naphtha (Erdöl), mit Wasserstoff behandelte schwere, zentrales Nervensystem, Neurotoxizität, MAK-Wert, maximale Arbeitsplatzkonzentration, Spitzenbegrenzung, naphtha (petroleum), hydrotreated heavy, central nervous system, neurotoxicity, MAK value, maximum workplace concentration, peak limitation

## Abstract

The German Senate Commission for the Investigation of Health Hazards of Chemi﻿cal Compounds in the Work Area (MAK Commission) summarized and re-evaluated the data for the derivation of the occupational exposure limit value (maximum concentration at the workplace, MAK value) for naphtha (petroleum), hydrotreated heavy [64742-48-9]. The critical effect is preclinical neurotoxicity. The results of a 13-week study with an isoparaffinic product in rats and a behavioural toxicity study in volunteers do not contradict the previous MAK value of 50 ml/m^3^. The assignment to Peak Limitation Category II with an excursion factor of 2 is retained. Additionally, there are no experimental data showing that juvenile animals are more sensitive to naph﻿tha-induced neurotoxic effects than adults. Therefore, there is no evidence that would require changing the previous categorization from Pregnancy Risk Group D to Pregnancy Risk Group B (suspected prenatal toxicity).

**Table:** 

**MAK-Wert (2009)**	**50 ml/m^3^ (ppm) ≙ 300 mg/m^3^**
**Spitzenbegrenzung (2009)**	**Kategorie II, Überschreitungsfaktor 2**
	
**Hautresorption**	**–**
**Sensibilisierende Wirkung**	**–**
**Krebserzeugende Wirkung**	**–**
**Fruchtschädigende Wirkung (2009)**	**Gruppe D**
**Keimzellmutagene Wirkung**	**–**
	
**BAT-Wert**	**–**
	
Synonyma	Kohlenwasserstoffe, paraffinische und naphthenische, C_6_–C_13_, Siedebereich 65–230 °C, aromatenfrei Stoddard Solvent IIC^a)^ „White Spirit“, entaromatisiert (White Spirit Type 3)^a)^
CAS-Nr.	64742-48-9
**1 ml/m^3^ (ppm) ≙ 6 mg/m^3^**	**1 mg/m^3^ ≙ 0,167 ml/m^3^ (ppm)**

^a)^ enthält nur höhersiedende Komponenten

In diesem Nachtrag wird u. a. der MAK-Wert geprüft, da zwei Studien an Menschen und Tieren durchgeführt wurden, die für die Begründung von 2009 (Hartwig 2010) nicht vorlagen, aber im Nachtrag zu Destillate (Erdöl), mit Wasserstoff behandelte leichte (CAS.-Nr. 64742-47-8; Hartwig und MAK Commission 2016) beschrieben wurden und auch für mit Wasserstoff behandelte schwere Naphtha (Erdöl) relevant sind.

White Spirit Type 3 (entaromatisierter „White Spirit“) entspricht der CAS-Nummer 64742-48-9, ist aber nur eine Teilmenge, da die wesentlichen Bestandteile von White Spirit Type 3 C_9_–C_11_-Kohlenwasserstoffe sind.

Die Daten im ECHA-Registrierungsdossier (ECHA 2023) zur CAS-Nummer 64742-48-9 basieren dagegen fast ausschließlich auf Studien mit bleifreiem Benzin (CAS-Nummer 86290-81-5 und CAS-Nummer 8006-61-9) mit einem Siedebereich von 30–260 °C.

## Erfahrungen beim Menschen

Die NOAEC bei 4-stündiger Exposition von Probanden (Lammers et al. 2007 in Hartwig 2010) gegen aromatenhaltigen „White Spirit“ beträgt ca. 600 mg/m^3^ (100 ml/m^3^). Neuropsychologische Tests ergaben bei dieser Konzentration keine bedeutenden Beeinträchtigungen und keine lokalen Effekte. Bei 30-minütiger Exposition gegen ein aromatenhaltiges Gemisch war die NOAEC für in Leistungstests gemessene Wirkungen auf das Zentralnervensystem (ZNS) 400 ml/m^3^ und die LOAEC 680 ml/m^3^ (Gamberale et al. 1975 in Hartwig 2010).

Mit „low aromatic White Spirit“ und aromatenhaltigem „White Spirit“ wurden bis zur höchsten getesteten Konzentration von 300 mg/m^3^ (50 ml/m^3^) in einer Probandenstudie keine bedeutenden verhaltenstoxischen Effekte bei 4-stündiger Exposition festgestellt (Hartwig und MAK Commission 2016; Juran et al. 2014).

## Tierexperimentelle Befunde und In-vitro-Untersuchungen

In einer Studie aus dem Jahr 1980 mit einem aromatenfreien Produkt (C_10_–C_12_-Isoparaffine, 170–187 °C) wurden 18 männliche und 18 weibliche Wistar-Ratten (10–13 Wochen alt) pro Gruppe 13 Wochen lang an fünf Tagen pro Woche, sechs Stunden pro Tag gegen 0, 359, 737 oder 1444 ml/m^3^ Ganzkörper-exponiert (Carrillo et al. 2013; Shell 1980). Die genaue Beschreibung der Studie und die Bewertung der Ergebnisse ist nachfolgend aus Hartwig und MAK Commission (2016) entnommen:

„Die Anämie ist nur sehr gering und auch nicht deutlich konzentrationsabhängig und tritt nur bei männlichen Tieren auf. Aber eine leichte Anämie bei männlichen Ratten wurde auch bei Exposition gegen andere aliphatische Lösemittel gefunden (Carrillo et al. 2013). Als Erklärungsmöglichkeit wurde von den Autoren die normale Variation der Blutparameter diskutiert. Sie weisen darauf hin, dass diese Veränderungen konsistent mit solchen sind, die als „Anemia of chronic disease“ beschrieben wurden. Solche Veränderungen wurden oft in Studien mit hohen Dosen beobachtet und sind toxikologisch nicht relevant (Car et al. 2006). Auch in der Studie mit Stoddard Solvent IIC (C_10_–C_14_, Aromatenanteil maximal 1 %; NTP 2004) kam es ab 1100 mg/m^3^ bei männlichen F344-Ratten nach drei Monaten zu leichter Anämie, nicht jedoch bei weiblichen Tieren und bei männlichen und weiblichen B6C3F1-Mäusen. Stoddard Solvent IIC führte ebenfalls nur bei männlichen F344-Ratten zu alpha-2u-Nephropathie. Daher ist anzunehmen, dass die Anämie ein Sekundäreffekt ist. NTP wertete die Anämie-Befunde als nicht toxikologisch relevant (NTP 2004). In der hohen Konzentrationsgruppe traten nur bei den männlichen Ratten verringerte Leukozytenzahlen auf. Die Leukozytenzahlen unterliegen innerhalb der Rattenstämme einer großen Schwankungsbreite (Caret al. 2006). Außerdem sinkt die Leukozytenzahl bei Ratten im Lauf des Lebens (NTP 2004), so dass das Anfangsalter der Tiere bei der Bewertung dieses Effekts von Bedeutung ist. Mit anderen aliphatischen Lösemittelgemischen wurde überwiegend kein Effekt auf die Leukozytenzahl beobachtet. In der hohen Konzentrationsgruppe trat nach der Exposition Lethargie auf. Dies ist ein adverser Effekt auf das ZNS. Zum erhöhten Lebergewicht führen die Autoren an, dass keine histologischen Befunde in der Leber und keine Aktivitätserhöhung der Aspartat-/Alanin-Aminotransferase festgestellt wurde, dies also ein adaptiver Effekt ist. Möglicherweise liegt diesem eine Induktion fremdstoffmetabolisierender Enzyme zugrunde, was jedoch nicht untersucht worden ist. Eine 40%ige Erhöhung des absoluten Lebergewichts in der Gruppe mit der hohen Konzentration durch Enzymindukti﻿on wird von der Kommission als unerwünscht gesehen, da diese in Stoffwechselvorgänge eingreifen kann. Die 14%ige (falsch im Text: 13%ige) Erhöhung in der mittleren Konzentrationsgruppe kann dagegen als NOAEC betrachtet werden. Die Inzidenzen der entzündlichen Infiltrate in der Lunge der männlichen Tiere waren konzentrationsabhängig erhöht. Die Autoren weisen darauf hin, dass das häufig bei den Ratten des Testlabors auftrat, und dass die Inzidenz ebenso hoch gewesen sei wie bei Kontrolltieren. Daten hierfür wurden aber nicht gezeigt. Das absolute Nierengewicht der weiblichen Ratten war in der hohen Konzentrationsgruppe ohne histologische Befunde erhöht. Die Nierenbefunde bei männlichen Tieren hängen mit der alpha-2u-Nephrotoxizität zusammen, sind also nicht für den Menschen relevant. Insgesamt treten verschiedene Effekte in der höchsten Konzentrationsgruppe auf, die von der Kommission als substanzbedingt und advers gewertet werden, so dass 1444 ml/m^3^ als LOAEC und 737 ml/m^3^ (falsch im Text: 759 ml/m^3^) als NOAEC anzusehen sind.“

## Bewertung

Kritischer Effekt von mit Wasserstoff behandelten schweren Naphtha (Erdöl) ist die Wirkung auf das zentrale Nervensystem (Hartwig 2010).

**MAK-Wert. **In einer 13-Wochen-Inhalationsstudie an Wistar-Ratten mit einem aromatenfreien Produkt (C_10_–C_12_-﻿Isoparaffine, 170–187 °C) wurde als empfindlichster Effekt erhöhtes Lebergewicht ab der niedrigsten Konzentration von 359 ml/m^3^ gefunden, jedoch ohne histologische und klinisch-chemische (Aspartat-/Alanin-Aminotransferase) Effekte. Bei 359 ml/m^3^ war das Lebergewicht um weniger als 10 %, bei 737 ml/m^3^ um 14 % und bei 1444 ml/m^3^ um ca. 40 % erhöht. Bei der höchsten Konzentration zeigten sich nach der Exposition Wirkungen auf das zentrale Nervensystem in Form von Lethargie (Carrillo et al. 2013; Shell 1980). Die systemische NOAEC ist 737 ml/m^3^. Aus Humanstudien wurden deutlich niedrigere NOAEC und LOAEC erhalten, daher wird der MAK-Wert aus den Human- und nicht aus den Tierstudien abgeleitet:

Aus den akuten verhaltenstoxikologischen Humanstudien (4-Stunden-NOAEC 100 ml/m^3^; 30-Minuten-NOAEC 400 ml/m^3^, LOAEC 680 ml/m^3^) wurde für mit Wasserstoff behandelte schwere Naphtha (Erdöl) ein MAK-Wert von 50 ml/m^3^ festgelegt (Hartwig 2010). Der MAK-Wert berücksichtigt auch das erhöhte Atemvolumen am Arbeitsplatz und eine Kumulation über die Arbeitswoche (Hartwig und MAK Commission 2016). Die neue Probandenstudie von Juran et al. (2014) mit der 4-Stunden-NOAEC von 50 ml/m^3^ widerspricht dem nicht, da keine höhere Konzentration getestet wurde. Der MAK-Wert wird daher beibehalten. Der Siedebereich von 65–230 °C ist niedriger als der von mit Wasserstoff behandelten leichten Destillaten (Erdöl) (150–290 °C), daher ist die Möglichkeit einer Aerosolbildung geringer und es wird, anders als bei diesen, für mit Wasserstoff behandelte schwere Naphtha (Erdöl) kein MAK-Wert für die alveolengängige Aerosolfraktion festgelegt.

**Spitzenbegrenzung. **Wegen der systemischen Wirkung bleiben mit Wasserstoff behandelte schwere Naphtha (Erdöl) in die Kurzzeitwert-Kategorie II eingestuft.

Aufgrund der berechneten initialen Halbwertszeit im Gehirn von unter zwei Stunden (Hartwig 2010) werden akute ZNS-Effekte rasch einsetzen und abklingen, weshalb ein Überschreitungsfaktor von 2 zugeordnet wird. Die dadurch zulässige Kurzzeit-Konzentration von 100 ml/m^3^ hat sich bei Probanden als nicht lokal reizend erwiesen und liegt unterhalb der 30-Minuten-NOAEC von 400 ml/m^3^ (siehe „MAK-Wert“).

**Fruchtschädigende Wirkung. **Für mit Wasserstoff behandelte schwere Naphtha (Erdöl) ist der MAK-Wert von 50 ml/m^3^ (300 mg/m^3^) reevaluiert und bestätigt worden. Die kritischen Effekte für die MAK-Wert-Ableitung sind lokale Wirkungen sowie Neurotoxizität. Die Studien zur pränatalen Entwicklungstoxizität bei Ratten bleiben bis 900 ml/m^3^ (5000 mg/m^3^) ohne Effekte (C_9_–C_13_-Gemisch). Im Jahr 2009 ist Naphtha der Schwangerschaftsgruppe D zugeordnet worden, weil zur Entwicklungsneurotoxizität keine validen Daten vorliegen (Hartwig 2010). Es liegen keine experimentellen Daten vor, die zeigen, dass juvenile Tiere empfindlicher für Naphtha-induzierte neurotoxische Effekte sind als adulte Tiere. Daher lässt sich ein Verdacht auf eine entwicklungstoxische Wirkung nicht begründen.

**Krebserzeugende Wirkung. **Es liegen keine neuen bewertungsrelevanten Studien vor. Der Stoff wird daher weiterhin nicht in eine Kategorie für Kanzerogene eingestuft (siehe Hartwig 2010).

**Keimzellmutagene Wirkung. **Es liegen keine neuen bewertungsrelevanten Studien vor. Der Stoff wird daher weiterhin nicht in eine Kategorie für Keimzellmutagene eingestuft (siehe Hartwig 2010).

**Hautresorption. **Der Stoff ist aufgrund einer In-vitro-Studie mit Rattenhaut nicht mit „H“ markiert (siehe Hartwig 2010). In einem Review wird bestätigt, dass die dermale Exposition gegen Erdöl-Substanzen gering ist und unter realen Anwendungsbedingungen kein Gesundheitsrisiko birgt (Jakasa et al. 2015). Der Stoff wird daher weiterhin nicht mit „H“ markiert.

**Sensibilisierende Wirkung. **Zur hautsensibilisierenden Wirkung beim Menschen liegen nach wie vor keine Daten vor. Ferner stehen keine neuen tierexperimentellen Untersuchungen oder Daten aus tierversuchsfreien Alternativverfahren zur Verfügung. Zur atemwegssensibilisierenden Wirkung liegen keine Daten vor. Der Stoff wird daher weiterhin weder mit „Sh“, noch mit „Sa“ markiert.
